# Supplementary material for: A UMLS-based spell checker for natural language processing in vaccine safety
Source: BMC Med Inform Decis Mak. 2007 Feb 12;7:3. doi: 10.1186/1472-6947-7-3 (PMC1805499; doi:10.1186/1472-6947-7-3)
Supplement: Additional file 13 — RAP application directory. Sets up directory for RAP (RDF application for PHP) source code [file 1472-6947-7-3-S13.gz › rap/doc/phpdoc/li_model.html]

xml version="1.0" encoding="iso-8859-1"?


model

Description
:   Class trees  
    Index of elements  
    Todo List

Classes
:   BlankNode
:   DbModel
:   DbStore
:   Literal
:   MemModel
:   Model
:   ModelFactory
:   Node
:   Resource
:   Statement

Files
:   Blanknode.php
:   DbModel.php
:   DbStore.php
:   Literal.php
:   MemModel.php
:   Model.php
:   ModelFactory.php
:   Node.php
:   Resource.php
:   Statement.php

phpDocumentor v 1.3.0RC3
